# “Vicious, Aggressive Bird Stalks Cyclist”: The Australian Magpie (*Cracticus tibicen*) in the News

**DOI:** 10.3390/ani6050029

**Published:** 2016-04-26

**Authors:** Kitty van Vuuren, Scott O’Keeffe, Darryl N. Jones

**Affiliations:** 1 School of Communication & Arts, The University of Queensland, Brisbane 4072, Australia; 2 Environmental Futures Research Institute, Griffith University, Brisbane 4111, Australia; m.s.okeeffe@optusnet.com.au (S.O.); d.jones@griffith.edu.au (D.N.J.)

**Keywords:** Australian Magpie, human-animal conflict, urban ecology, newspapers, framing, *Leximancer*

## Abstract

**Simple Summary:**

This article explores the role of print media in reporting the conflict between the Australian Magpie (*Cracticus tibicen*) and human populations in Australia. The results indicate that this issue is primarily covered during the spring “swooping” season in the regional and suburban press.

**Abstract:**

The Australian Magpie (*Cracticus tibicen*) is a common bird found in urban Australian environments where its nest defense behavior during spring brings it into conflict with humans. This article explores the role of print media in covering this conflict. *Leximancer* software was used to analyze newspaper reports about the Australian Magpie from a sample of 634 news stories, letters-to-the editor and opinion pieces, published in newspapers from around Australia between 1 January 2010 and 31 December 2014. The results confirm that stories about these birds are primarily published in the daily regional and weekly suburban press, and that the dominant story frame concerns the risk of “swooping” behavior to cyclists and pedestrians from birds protecting their nests during the spring breeding season. The most prominent sources used by journalists are local and state government representatives, as well as members of the public. The results show that the “swooping season” has become a normal part of the annual news cycle for these publications, with the implication that discourse surrounding the Australian Magpie predominantly concerns the risk these birds pose to humans, and ignores their decline in non-urban environments.

## 1. Introduction

The Australian Magpie (*Cracticus tibicen*) is a common native bird found in parks, sports fields, schoolyards, and nature strips in nearly every Australian city, town and region south of the Tropic of Capricorn [1]. The species is among Australia’s top 10 favorite birds according to a poll of 8000 people, conducted by *Birdlife Australia* magazine in 2013 [2]. However, magpies are also despised and feared because of a tendency for certain individual birds to swoop and attack some people during the spring breeding season, a problem that has become prevalent in the suburbs of many Australians towns and cities [3]. According to Jones [1], 85 percent of residents in the city of Brisbane claim to have experienced an attack during their lives. Sometimes these attacks result in permanent injuries, or even death. For example, in August 2010, west of Ipswich, Queensland, the family of a 12-year-old boy who died after running into the path of a car, believed a magpie attack was responsible for the accident [4]. Tragically, significant numbers of people suffer serious eye injuries annually as a result of these attacks [5]. Reports of the severity of such attacks have encouraged people to demand that authorities, including wildlife agencies and local governments, deal with problem birds, which inevitably sees residents who want the birds removed opposed by those who want them protected [3].

Jones [1] suggests that human–magpie conflicts are amplified, exaggerated by the way the popular media cover stories about the species. Indeed, media scholars claim that patterns of media attention rarely parallel “the actual trajectory of any particular threat” [6]. This article applies content analysis to explore the role of Australian news media in human–wildlife conflict, using the Australian Magpie as a case study. Although most people have some personal encounter with birds, we understand them through human experiences, language, emotions, and interpretations located within social, historical and cultural contexts [7]. Public knowledge and understanding of birds and other wildlife increasingly comes from popular culture messages from film, television, advertising, and news media. Our study provides insights into how the Australian print news media *frame* the discourse surrounding the Australian Magpie, referring to the language used to define a particular problem, its causes, preferred solutions and moral perspectives [8]. We chose print media because these offer an easily available, permanent record of their content compared to broadcast media, and newspaper markets are more diverse compared to other mainstream media [9]. We identify the most dominant themes that typify the reporting of magpies in the media and ask if there are differences between media markets and geographical locations; and who speaks for or against these birds. We present the results from a qualitative analysis of Australian newspaper stories and letters to the editor about the Australian Magpie (hereafter “magpie”) published between 1 January 2010 and 31 December 2014. Articles were gathered from the Factiva database, and analyzed using *Leximancer* (Version 4.0) text analysis software [10]. Before turning to the method and the analysis, we first present a summary of relevant literature addressing the role of news media in human–wildlife conflicts.

## 2. Wildlife in the News

In Australia native wildlife species are legally protected in all states with exceptions where wildlife are thought to pose a risk to property or human safety, such as damage to crops and risks of collision with aircraft; duck hunting is also permitted. Temby notes that management approaches tend to be inconsistent and premised on an inadequate understanding of the situation, and are reactive and based on “short-term alleviation of complaints” [11] (p. 29). He claims that the ways in which wildlife issues are reported in the media may contribute to this situation.

Since there is little Australian research that examines representation of wildlife in the media [9], we rely on a more established tradition of wildlife and media research from North America, the United Kingdom and Europe. Past research indicates that media coverage of wildlife is popular. Several studies from the 1990s in the UK and US found that wildlife stories make up between 20 and 25 percent of all environmental stories on television [12,13]. There are no recent comparative studies of the volume and extent of wildlife coverage in broadcasting, online or in newsprint and even less is known about wildlife representations in the regional and suburban press. Nevertheless, Corbett [12] points out that wildlife coverage is popular and people may identify more closely with animals than with other aspects of the environment such as plants or geography. The success of dedicated international television channels, such as *Animal Planet* and *Discovery Channel*, further points to the popularity of this genre. However, news coverage of wildlife (as distinct from entertainment formats) tends to be treated as “soft news” implying that it is of lesser importance [14]. Wildlife news stories rarely reach the headlines except when these threaten or cause death (such as shark or crocodile attacks), or where they symbolize the impacts of disasters such as oil-spills and habitat loss.

News is the result of professional media production practices [15]. Media organizations, like other institutions, produce, circulate, distribute, consume, and reproduce their own preferred meanings, and therefore can negotiate the meaning of messages in society more generally [16]. Media have a central role in reinforcing dominant voices and portraying these as “common sense”, while ignoring others. It follows that media must be treated with caution when used for public education purposes. Past research suggests communication activities from, for example, wildlife managers that seek to educate the public can sometimes have short-term effects, but most communication campaigns fail [17]. In his discussion of environmental advertising, Heberlein [18] claims that advertising and directed media coverage does not change behavior. He stresses that media content is effective when it resonates with pre-existing beliefs, attitudes, experiences and behaviors.

To journalists a story must be “newsworthy”, referring to its ability to appeal to their readers and audiences. They invoke so-called “news values”, an ambiguous and arbitrary concept, to identify and select items that are considered newsworthy. Common news values include stories about powerful people or organizations (the *power elite*) and the famous (*celebrity*); stories that *entertain* (including stories about animals), or that have an element of *surprise*; *bad news* focusing on conflict or tragedy, or *good news* concerning rescues or cures; stories that are significant to a large number of readers (*magnitude*), and are perceived to be *relevant* to an audience; stories that *follow up* existing news topics; and stories that fit a news organization’s own *agenda* [15] (p. 43). A news story can fall into several of these categories.

The news value of “relevance”, referring to stories selected on the basis of their proximity to a news organization’s readership, was identified by Bendix and Liebler [19] in a study measuring the geographic variation of news coverage in major daily U.S. newspapers over the conflict concerning the preservation of Spotted Owls (*Strix occidentalis caurina*) and old-growth forest in the Pacific Northwest. They found that news coverage of the issue decreased with geographic distance, reflecting the importance of the conflict to communities whose livelihoods depend on logging. Jacobsen *et al.* [20] found a similar trend in Florida, U.S., where local newspapers published significantly more news about the Florida Panther (*Puma concolor* *coryi*) than statewide newspapers. Results from a “uses and gratification” study of information-seeking habits among residents in New York, by Loker *et al.* [17], also found that many people relied on the local press, with its focus on local issues rather than television or radio, which cater for larger, more dispersed audiences, for news about problem White-tailed Deer (*Odocoileus virginianus*).

Other studies have revealed that the news value of “bad news” tends to focus on animal-human conflict. Gerber *et al.* [21] conducted a study of animal representations in the Swiss media over a 30-year period and found that stories about undesirable animals dominated the text corpus. Related to the “bad” news value is the “risk frame”, referring to perceived risks to humans from animals such as disease, injury, damage to property and aesthetics [21,22]. The “jobs *versus* the environment” frame, identified by Bendix and Liebler [19] also conforms to this news value.

Several studies suggest these frames are underpinned by a more fundamental frame, the so-called “nature/culture divide” that assumes a clear spatial boundary between humans and wild animals [21,22,23,24,25]. Animals that transgress this boundary are considered problematic requiring management interventions. However, several studies have shown that media coverage that amplifies the risks to humans from wild animals does not necessarily lead to increased public concern. Gore *et al.* [24] conducted a pre- and post-survey of residents concerning a human fatality from a black bear attack in New York State, which found that despite increased media coverage, the perception of risk before and after the incident remained stable. Prior to the incident respondents believed such events were rare, and their views had not changed following the attack [24]. This study acknowledges the view that media coverage does not parallel actual events, nor that there is a direct relationship between media content and people’s beliefs, attitudes and behavior.

Other factors that shape the construction of news include commercial pressures and production routines including time constraints to meet deadlines, and reliance on the “information subsidy”, referring to the use of media releases and other public relations activities from government and corporate sources [15] (p. 59). Several studies of environmental news have shown that journalists tend to favor sources from government, politics and the business community [12,26]. To meet the demands of commercial news media organizations, time-poor journalists tend to report the views of those most easily sourced such as government agents and corporations; rarely do they report the opinions of scientists, naturalists, conservation organizations, or private individuals.

Reliance on information subsidies can lead to the dominance of particular frames favored by the more powerful institutions that have the resources to produce ready-made media content. Entman explains that the process of framing involves the selection and highlighting of “some aspects of a perceived reality” to “make them more salient”, while downplaying others [8] (p. 52). Routine journalism practices contribute towards framing news by simplifying facts and reconstructing events in terms that are familiar to readers [15]. For example, complex issues are simplified by taking an “episodic” approach that focuses on specific events or individuals, rather than a “thematic” approach where issues are examined in-depth and in their contexts [19,22,27]. Environmental issues are often presented as adversarial; as “seemingly irreconcilable conflicts between neatly defined, diametrically opposed groups” [28] (p. 24), a practice that reduces a complex issue to two contrasting positions and thus satisfies the “bad” news value. Perceptions of wildlife can also be influenced by the frequency of media coverage (corresponding to the news value of “follow-up”) [17,24]; the tendency to interpret new stories by anchoring these to past events [21]; and repetition of keywords, phrases and imagery within stories [22] that “fix” common sense understandings of an issue [29]. Through these practices, journalism sets the public policy agenda—telling people what to think about [30]—and transmits and emphasizes dominant and “ideologically loaded” meanings that result in stabilizing socio-cultural structures [15].

To shed light on the role of media in human-wildlife conflicts and management issues, our analysis seeks to answer the following research questions: what are the main themes in the print media that typify news coverage of the Australian Magpie, and how are these themes “framed”? When, and in which states and markets are stories about magpies most likely to be reported? Who are the main sources of news for magpie stories in the print media? In the next section we briefly describe the method, specifically the qualitative computer-assisted software used to conduct the analysis.

## 3. Method and Sample

*Leximancer* (Version 4.0, Leximancer Pty Ltd., Brisbane, Australia) is a software application that uses word co-occurrence statistics to extract a list of “concepts” from an input text. “Concepts” are “collections of words that generally travel together throughout the text” [10] (p. 10). The software gives weightings to words associated with a concept depending on how frequently they occur together. As well as detecting a concept in the text, the software also determines the frequency of co-occurrence between concepts. Data is grounded by the input text and the software can achieve levels of reliability and repeatability that are difficult to achieve with hand-based methods such as an analyst’s codebook [31]. To generate these statistics, *Leximancer* uses “text blocks” as the unit of analysis. In this study we used the setting of one sentence per block, to take into account the presence of very short reports. The software identifies discrete concepts in the text blocks and generates a thesaurus with a ranked list of terms for each concept. A text block can generate one or more concepts. The researcher can also access the text manually to access the data source [10].

The software generates several forms of analytical output, including a two-dimensional map that clusters concept nodes into coherent sections. The larger the size of a node the more prominent the concept is within the data. Nodes are grouped near other nodes to generate themes, based on conceptual relationships. The importance of themes is indicated with a ”heat-map” with red indicating the most important theme, and blue the least important. The concept map can also display a spanning tree that shows the most likely connections between concepts. The map is supplemented with a “Concepts Tab” that displays a rank-order list of concept frequencies in the text corpus. The analyst can also “tag” the data to generate categories to enable comparisons. *Leximancer* generates rank-ordered concept lists that indicate the strength of association between concepts and tags. In this study several concept maps were generated and tagged to indicate which states are most likely to report on magpies; and the month of publication to show the annual news cycle pertaining to magpie reports. Each concept map is supplemented by additional analytical output generated by *Leximancer*, including a detailed quantitative “Dashboard” report, which shows where concepts are ranked for each category. The “prominence” score combines a concept’s strength and frequency scores using Bayesian statistics and is a measure of the correlation between a category and the concepts attributed to it [10]. The frequency score is on a log scale and represents the conditional probability that a text extract (concept) comes from a particular category. Strength is the reciprocal conditional probability that a concept comes from that category. Strong concepts distinguish a category from others, regardless of whether concepts are mentioned frequently.

All newspaper articles about the Australian Magpie were collected from all Australian newspapers listed in the *Factiva* database between 1 January 2010 and 31 December 2014. Search terms included “magpie”, and “bird”, but not “Collingwood”, “football”, “disease”, “geese”, “goose”, and “lark” (because of potential confusion with sporting teams and other bird species). The search generated a total of 634 articles published in 204 Australian publications, including nine national publications, 16 state-wide newspapers that publish daily, 118 regional newspapers that publish daily or weekly, and 61 suburban newspapers that publish weekly, fortnightly or monthly.

## 4. Results

### 4.1. Locations, Concepts and Themes

Table 1 presents the frequency of stories published by year and state. It indicates that the Queensland press publishes more stories about magpies than any other state, over a third of the total (37.5%, *n* = 634). The New South Wales press published more than a quarter (28.9%) and the Victorian press published 20.2 percent of the total number of stories. Newspapers in these three states together published 86.8 percent of all stories about magpies during the sample period, indicating that stories about magpies are most relevant to readers in Australia’s eastern states, which is where most Australians live.

**Figure 1:**
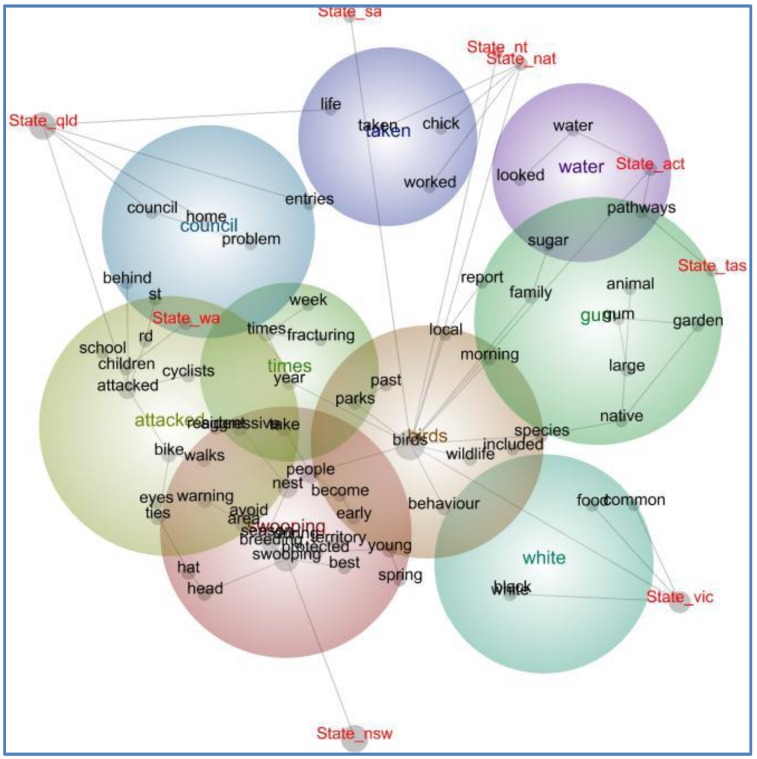
Concept map of newspaper coverage of the Australian magpie by state, 2010–2014 (*n* = 634).

The 634 articles included in the computer-assisted analysis generated 9817 concept blocks and 68 concepts. Figure 1 presents the concept map generated by *Leximancer*, including manually added tags for state categories. The concept “magpie” was removed from the computer analysis since it links to all the categories, most of the concepts, and overlapped with the concept “birds”. Its removal did not greatly affect the results. However, the concept “birds” was included, since its removal resulted in minor concepts and themes present in states outside Queensland, New South Wales and Victoria, being removed from the analysis.

The concept map clearly shows that “swooping” is the most important theme in the text corpus; it yielded 1245 “hits” (*i.e.*, frequency) surpassed only by “birds” (2226 hits). This theme comprises the concepts swooping, nest, young, protected, breeding, aggressive, season, spring, people, area, warning, avoid, territory, hat and head. Stories associated with this theme are typically framed in terms of risks and hazards associated with aggressive magpies during the spring breeding season and provide advice to pedestrians and cyclists on how to avoid and protect themselves from attacks from aggressive birds. The spanning tree shows that themes concerning magpie swooping behavior and the risk to children, cyclists and pedestrians are closely associated in Queensland, New South Wales, Victoria and Western Australia.

The second most important theme is “birds” comprising the concepts *birds, wildlife, parks, species,* and *behaviour.* The spanning tree shows that all the state categories except Queensland, New South Wales and Western Australia are directly linked to the “birds” theme. This theme is less concerned with the magpie’s behavior during the breeding season, although it is connected to it, but also includes stories about wildlife rescue and care, bird identification and bird feeding.

The third most important theme is “attacked”, comprising the concepts *attacked*, *ties, eyes, walks, bike, cyclists, children* and *school*. It is closely associated with the “swooping” theme, especially of children on their way to and from school, and includes articles that explain how pedestrians and cyclists can protect themselves. For example: “Cyclists have led the pack with anti-swooping strategies, attaching cable ties to their helmets, creating an odd but effective method of warding off swoopers” [32]. This theme connects directly with the Queensland and Western Australian categories, indicating its importance in those states.

Although less strong, the theme “council” connects with the *State_qld* category, indicating that in the state of Queensland text excerpts referring to actions taken by local governments (“councils”) in response to aggressive magpies are more prominent. The remaining themes are more general-interest related and include stories with descriptions of magpies, their presence in parklands or gardens, or stories about bird rescues.

Table 2, derived from *Leximancer*’s Dashboard report shows that the closely associated concepts “attacked” and “aggressive” (located in the themes “swooping” and “attacked”) are most prominent in the Queensland press, while “swooping” is more prominent in the Victorian press. However, all states mention “swooping” in the top ten ranked concepts, except for Tasmania, where no concepts related to magpie swooping behavior are prominent (based on a single article retrieved from the *Factiva* search).

In summary, newspaper coverage of magpies is dominated by stories that frame magpie swooping as behavior that occurs during the breeding season and is a risk to cyclists and pedestrians. Cyclists and pedestrians can reduce the risk of being attacked by avoiding the area where the bird is breeding and by protective measures such as wearing a hat, sunglasses and cable ties on bicycle helmets. Where birds are hazardous, such as near schools, state and local authorities are expected to address the problem. Magpie stories are most newsworthy in Queensland, New South Wales and Victoria. Having established the states where coverage of magpies and the risk frame is prominent, we now examine in more detail which newspaper markets, “national”, “state”, “suburban” and “regional”, are most likely to publish stories about this bird.

### 4.2. Which Markets?

Table 3 indicates that news coverage about magpies is foremost a topic reported in regional markets (57.8% of all newspapers, and 60.4% of all stories), in newspapers located in larger regional centers such as Toowoomba, Grafton, Bendigo, and others. Many of these newspapers publish daily from Monday to Friday and usually also publish a Saturday edition. In the Australian Capital Territory (ACT), magpie stories are only reported in the state publication. Canberra, Australia’s capital city is located in the ACT, which is small in geographical size and population. The circulations of its state publications are comparable to that of regional newspapers in other states.

Stories about magpies are also likely to be found in the suburban press (29.9% of all papers and 26.3% of all stories), especially in Queensland. Most of these are weekly publications and usually distributed freely to households [33]. In Melbourne and Brisbane, the suburban press is dominated by subsidiaries of News Corp Australia (Surry Hills, Sydney, Australia) (Leader and Quest mastheads). A closer examination of the text corpus points to a high degree of syndication in suburban newspapers owned by these companies, with many stories republished in newspapers from nearby suburbs. For example, a story about a magpie attack on a resident riding his bicycle at Pine Rivers (north of Brisbane, Queensland), was published in twelve suburban weekly newspapers owned by Quest News, in October 2014. As seen in the excerpt below, the story suggests “many” people are at risk.

The bird swooped several times, and Mr. Habib lost his balance and came off his bike, fracturing his shoulder. His story is one of many from people who have been injured, or at least rattled, by territorial magpies this year (Albert and Logan News, Caboolture Shire Herald, Pine Rivers Express, City South News, City North News, Redcliffe and Bayside Herald, Southern Star, Wynnum Herald, South East Advertiser, Westside News, South West News, North West News.).

Table 4 presents the ten most prominent concepts for each market category. Although not unique to the suburban press, the concept “swooping” is most prominent in that category, while it ranks lower in all other categories. In the regional press the concept “breeding” ranks higher than “swooping” indicating that in these newspapers attention is drawn to the fact that magpies are swooping because they are breeding. In the suburban press “swooping” and “bike” rank higher pointing to greater prominence of stories about magpie attacks on people riding bicycles. National and state newspapers appear less concerned with swooping and more interested in other aspects of the species that are more likely to appeal to their larger and more general readerships.

In Table 4, the concept “area” in the regional and suburban press points to the more active role taken by these newspapers in identifying and mapping locations where magpies are causing concern. For example, readers of the suburban newspaper *Albert and Logan News*, are asked to report he locations where magpies are swooping, so that these can be included in the online Magpie Map published by the paper’s parent company Quest News (“Vigilant readers wary of magpie menace” (17 September 2010), *Albert and Logan News*, p. 6, Queensland). Similar approaches have also been adopted in other states (“Watch out it’s magpie season” (29 September 2010), *Eastern Courier Messenger,* p. 19, South Australia; “*Attacks from the air, Birds zero in on unsuspecting cyclists*” (14 September 2010), *Knox Leader*, p. 1, Victoria).

### 4.3. Swooping Season

**Figure 2:**
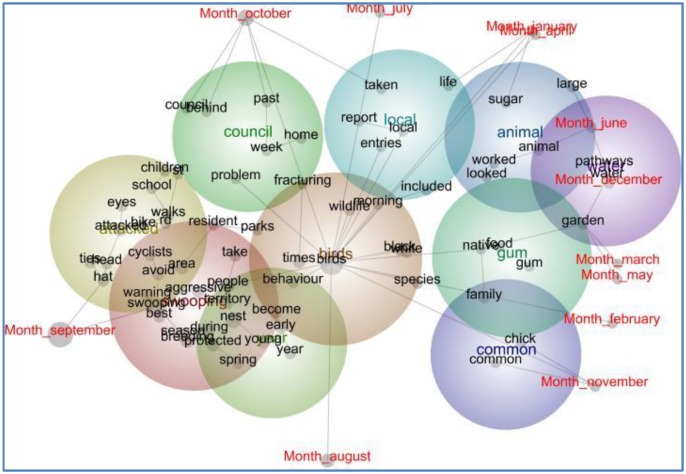
Concept map of newspaper coverage of the Australian Magpie by month, 2010–2014 (*n* = 634).

The concept map in Figure 2 shows that the concept “season” is grouped with the “swooping” theme, representing newspaper stories that identify a discernible “swooping season”. The spanning tree further shows that the concept “season” is connected to “breeding” and “protection”, also located in the “swooping” theme, as well as the theme “year”, comprising the concepts “spring”, “nest” and “young”. The concepts in Figure 2 have been tagged to indicate the time of year magpie stories are most likely to be published. It confirms that the themes “swooping”, “attacked”, “council”, and “birds” are most important during August, September, and October, coinciding with the spring breeding season, as illustrated by the following excerpts:

*SWOOPING season is upon us and Wyndham residents are being reminded to keep an eye out for magpies and other swooping birds*.

(“Seasonal loop is around with the swoops, so watch it”, 25 October 2011, *Wyndham Leader*, p. 3, Victoria)

*A VICIOUS magpie is terrorising a South Grafton street, drawing blood from its victims and virtually closing a popular park. In addition to its savagery, the magpie has also been attacking people in Riverside Dr(ive) for the past six weeks, well before the traditional swooping season*.

[34]

*TWEED residents will need to keep an eye out for black and white dive-bombers, with magpie season in full swing*.

[35]

The newspapers’ focus on magpie behavior during the spring season suggests it has been added to the annual event calendar that structures news coverage [33]. Of particular interest is a story published in the *Canberra Times* about the inclusion of the term “swooping season” in the Australian National Dictionary, further pointing to the normalization of understanding the Australian Magpie as a species that is a risk to humans during spring:

*The swooping season is upon us once again and, according to the Oxford English Dictionary (OED), we have The Canberra Times to thank for this wonderfully evocative term which will be included in the next edition of the Australian National Dictionary. An OED forensic word expert has traced the term back to the following: “The love/hate relationship between Canberra citizens and their magpies is of very long standing. The September–October swooping season has long been an accepted part of life here”. (Canberra Times, 30 November 1984)*.

(“Swooping season: a dictionary definition hatched in Canberra”, 4 September 2013, *Canberra Times*, p. 12)

### 4.4. Who Speaks: Sources

**Figure 3:**
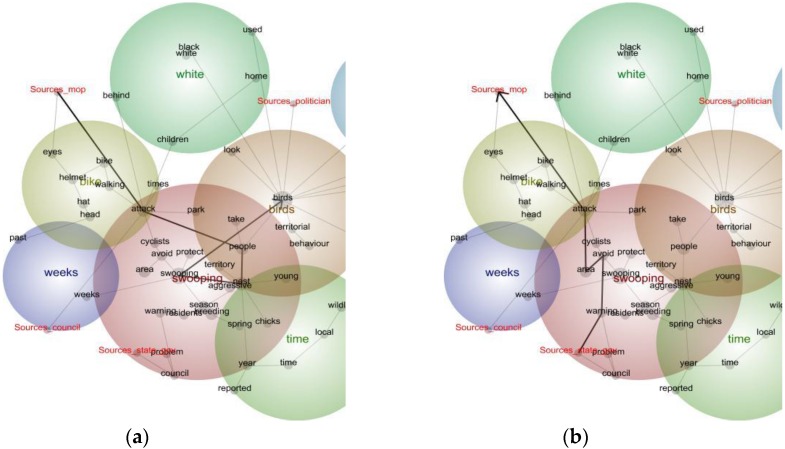
Concept map pathway analysis of newspaper coverage of Australian Magpies by Sources (*n* = 634): (**a**) pathway analysis, members of the public; (**b**) pathway analysis, state government sources.

Each story was coded (tagged) for up to three sources, including “member of the public” (MoP), “council” (local government); “state government” (including Parks and Wildlife Services), “scientists”, “birdwatcher”, “business”, vet (including the Australian Veterinary Association), “politician”, “wildlife carer”, “RSPCA”, “fauna control” (referring to business that remove problem wildlife). Figure 3 indicates that local (council) and state government sources, as well as individual members of the public are the most common sources cited in news stories about Australian Magpies. Figure 3a provides a “pathway analysis”, represented by the black arrows that indicate that members of the public in news stories are most likely to talk about magpies as birds that behave aggressively when they are nesting. Stories often include a brief response from a representative of the relevant state government department, local government, a vet, scientist, or medical doctor. Figure 3b indicates that news stories most often include state government sources, as well as from councils, who respond to members of the public with advice that cyclists and pedestrians avoid areas where magpies attack, and remind readers that this behavior will cease when breeding season ends.

Such stories typically follow a similar format: they open with a personal anecdote from a member of the public who has experienced an attack by a magpie while walking or cycling; followed by a response from the local or state government, and concluding with advice to avoid future attacks. For example:

*WARWICK has swung into swooping season with magpies protecting their young across the Rose City—resulting in a broken arm for one lady... Ambulance officers treated her at the seen* (*sic*) *before taking her to the Warwick Hospital... Queensland Parks and Wildlife Service Senior Ranger Adam Northam said the peak of breeding season was from late August to October. “During the breeding season, the best approach is to stay well clear of areas where magpies are known to be swooping, particularly the nesting tree… Avoid attacks: Don't provoke or harass as it makes them more defensive. Avoid areas where magpies are known to swoop. Find the bird and keep watching it when entering a magpie territory. If swooped on, don't crouch in fear or stop. Move on quickly but don't run…Wear a hat or carry an umbrella”*.

[36]

These stories tend to be “episodic” rather than “thematic” [27]. Coverage routinely focuses on the magpie’s aggressive behavior, and explains that it is related to breeding season, that not all birds do this, that the public can take personal steps to avoid being swooped, and that they are protected native fauna that can only be removed under a permit. Stories do not offer any additional information about the bird’s natural history or its conservation status.

The other prominent source category of interest presented in Figure 3 is “politicians”. One story published in the national newspaper, *The Australian*, reports on a magpie that local residents in Tweed Heads, New South Wales, wanted destroyed after it “drew blood” from a young girl. Police were asked to shoot the bird, but refused. The “rogue magpie became the talk of the country” and the story includes the views of two federal politicians:

*Local residents and conservationists pleaded with authorities to spare the bird’s life, with federal Finance Minister Penny Wong lending her support. Despite having been swooped as a child in the Adelaide Hills, Senator Wong said that hadn't turned her against magpies. “Maybe I’m a softie”, she told ABC Radio. “I just don’t want to think about killing a creature”. But opposition legal affairs spokesman George Brandis said kids came before magpies. “I will go for keeping people safe every time”, Senator Brandis said*.

[37]

Although animal-human conflict stories are less likely to be published in the national press, the quotes from prominent federal politicians illustrate the “power elite” news value that appeals to *The Australian’*s readership. However, in this source category, politicians are more likely to be locally elected city councilors:

*Richlands Councilor Milton Dick said areas of the south-west were seeing a steady rise in the number of magpie attacks each year… “With magpie season upon us, it is critical everyone, particularly kids going to and from school, are aware of the nesting habits so they are not attacked by magpies”, Cr Dick said*.

[38]

## 5. Discussion: Normalizing “Swooping Season”

This study explored the role of Australian newspapers in the management of conflict between humans and the Australian Magpie. In summary, the results suggest that this conflict has become “normalized” with “swooping season” being a part of the annual news cycle. News about magpie attacks is primarily published in Australian regional and suburban newspapers. The emphasis on personal anecdotes from residents about magpie attacks at particular locations in these markets, suggests regional and suburban editors consider such stories to be of greater relevance to these readerships, supporting similar research results from other countries [19,20]. News coverage of the magpie conforms to a risk frame [21,24] with the discourse focusing on the risks to pedestrians and cyclists from swooping attacks that can result in injury, and even death. However, although in the city of Brisbane, for example, 85 percent of residents may have experienced a magpie attack [1], in the only study examining magpie attacks on humans, Jones and Thomas [39] report that few magpies actually attack humans. Their 1999 study of 59 magpie breeding pairs in various locations around Brisbane, found that only 11 individual birds, all of them male, were found to be aggressive towards humans. By contrast, the Brisbane magpie map compiled by Quest News [40], based on anecdotal reader reports from the greater metropolitan region, suggest there were more than 150 magpie attacks during the 2015 season.

Our results indicate that members of the public are common news sources in stories about swooping magpies: both the suburban and regional press give preference to anecdotes from individual members of the public describing their experience of a magpie attack and the sometimes-serious consequences that result from this. This result departs from other studies of environmental news where members of the public are not usually a source for news [12,26].

Clearly, the newspaper stories included in this study draw attention to the Australian magpie when its behavior during breeding season routinely puts these birds in the “bad” category, especially in the suburbs where unsuspecting members of the public become “victims” of aggressive birds. Though less prominently, this discourse also proliferates in regional newspapers that service larger regional centers with urbanized environments similar to suburbia. This “genre” of stories has become an important part of the annual news cycle, although without the advertising market that accompanies other annual news events, such as Christmas, Easter, Mother’s Day, and so on [33]. Some newspapers, such as those from the Leader and Quest News groups use this annual event to promote their own role in providing a community service to their readers with the promotion of “magpie maps” to encourage their readers to provide details of attacks and locations of problem birds in the suburbs.

Framing magpie swooping season as an annual event and the risks associated with this behavior contributes to the common-sense understanding of the Australian magpie as a “problem” bird that demands responses from relevant government authorities [41]. The use of personal anecdotes in the news reports function to identify with the readership since most readers may themselves have experienced an attack [1]. Typically the personal anecdotes are augmented with the scientific and regulatory information, usually sourced from local and state government officers, and less so from scientists or veterinary professionals. Official responses usually include warnings and advice to cyclists and pedestrians about ways to avoid magpie territories. Where risks from swooping birds result in serious injuries, residents demand that problem birds be removed. The newspaper stories indicate that the current approach by wildlife managers is to educate the public about how to avoid magpie attacks, and only in the last resort are problem birds removed from their territory. In contrast to Temby’s claim [11], newspapers report a consistent message to readers on how to avoid risks from swooping magpies; an approach that has been recommended elsewhere [24].

With “swooping season” being a discernible part of the annual news cycle, and newspapers actively seeking stories from the public, it can be expected that complaints in the form of personal anecdotes will continue to be made, as they form part of the way in which these stories are framed. Such framing amplifies the perceived severity of magpie attacks and reinforces the preferred solutions to the annual magpie “problem”. However, the individualistic, episodic approach to news coverage of magpie attacks may hinder the formation of an identifiable stakeholder group that could demand long-term management approaches that may be harmful to the magpie’s conservation status. Instead, suburban newspapers in particular, appear to have adopted an advocacy role that on the one hand amplifies the perceived risk of magpie attacks to the general public, but on the other hand accepts and supports management approaches promoted by wildlife managers.

## 6. Conclusions

The current approach to news stories with its emphasis on risk, framing the magpie as perpetrator and cyclist or pedestrian as victim, together with episodic framing of attacks from individual birds on individual people [27], directs attention away from the Australian Magpie’s natural history, including its conservation status, which has been in decline along the east coast since 1999 [42], yet none of the newspaper articles in the corpus mention this.

Although the risk to humans attached to “swooping season” is the dominant discourse, other discourses do occur, but they are far less prominent. At other times during the year news coverage of magpies includes stories about rescues of individual birds, for example where these have been injured; or as part of bird counts undertaken by birdwatching groups; or as part of “nature” where they are part of the backdrop of a tourism destination. However, in these stories, the Australian Magpie’s conservation status is also ignored. Although urban magpie populations are increasing, readers are never informed about their significant decline nationally [42]. This creates the impression that the Australian Magpie is ubiquitous with the consequence that it becomes more difficult to convey the fact that the species is in decline. We suggest that the annual “swooping season” presents wildlife managers with an opportunity to alert the public to the Australian Magpie’s conservation status and its overall decline.

This study has drawn attention to the ways in which Australian newspapers represent the conflict between humans and the Australian Magpie, and we conclude that the press contributes towards normalizing a common-sense understanding of magpies as ubiquitous and a seasonal problem. Future studies, however, need to take into account the increasingly important role of online and social media in shaping people’s knowledge and perceptions about wildlife. Newspapers are themselves increasingly moving their content online (for example the Quest News magpie map) and there are several Facebook pages that encourage residents to report magpie attack locations (for example, “Whyalla magpie attack locations”; “Dubbo magpie attacks”; “The magpie that attacks Terry everyday must duy” (die); but see also “The Magpie Whisperer”, a Facebook page that celebrates this bird). One consequence of this trend is that audiences and readerships become fragmented, which has implications for media campaigns that aim to educate the public about wildlife issues. We emphasize that current research indicates that there is no direct relationship between media content and people’s views towards wildlife [24]. A survey of readers, especially in suburban and regional markets may shed further light on the sources for, and impacts of wildlife news on readers and the extent to which newspaper reports and other media influence people’s beliefs, attitudes and behavior.

Finally, the promotion of magpie maps by the media with their reliance on anecdotal reports does not offer an accurate measure of the actual risk to humans from magpies. More up-to-date surveys of aggressive behavior by Australian Magpies on a national scale are required to determine the true extent of the risk.

## Figures and Tables

**Table 1:** Frequency of newspaper stories, by year and state (2010–2014).

State	2010	2011	2012	2013	2014	Total	%
Qld	32	53	40	51	62	238	37.5
NSW	11	41	24	43	64	183	28.9
Vic	11	28	29	21	39	128	20.2
WA	0	2	0	3	18	23	3.7
ACT	0	2	1	5	13	21	3.3
SA	3	5	1	2	5	16	2.5
NT	1	2	0	1	0	4	0.6
TAS	0	0	0	0	1	1	0.1
National	3	6	1	7	3	20	3.2
Total	61	139	96	133	205	634	100.0

**Table 2:** Summary of ranked concepts for categories (States) associated with Australian Magpie stories.

State	1st Ranked Concept (Prominence)	2nd Ranked Concept (Prominence)	“Swooping” Ranking (Prominence)	“Attacked” Ranking (Prominence)	“Aggressive” Ranking (Prominence)
QLD	Attacked (1.5)	Aggressive (1.4)	8 (1.0)	1 (1.5)	2 (1.5)
NSW	Protected (1.4)	Breeding (1.3)	7 (1.1)	10 (0.9)	-
VIC	Wildlife (1.7)	Swooping (1.4)	2 (1.4)	10 (0.7)	-
WA	Ties (2.9)	People (2.5)	9 (1.0)	8 (1.1)	5 (2.0)
ACT	Garden (5.9)	Species (3.5)	10 (0.3)	-	-
SA	Year (1.2)	Hat (1.2)	10 (0.6)	-	-
NT	Parks (4.6)	Black (3.8)	10 (0.4)	9 (0.8)	-
TAS	Pathways (12.8)	Report (11.1)	-	-	-
National	Chick (6.1)	Species (2.1)	10 (0.3)	9 (0.7)	-

**Table 3:** Frequency of publications and stories of the Australian Magpie by market, and state, 2010–2014.

**Market—Publications**										
**State**	**National**		**State**		**Regional**		**Suburban**		**Total**	
	***f***	***%***	***f***	***%***	***f***	***%***	***f***	***%***	***f***	***%***
Qld			1	6.3	43	36.4	16	26.2	60	29.4
NSW			3	18.8	47	39.8	19	31.1	69	33.8
Vic			5	31.3	21	17.8	17	27.9	43	21.1
WA			3	18.8	3	2.5	4	6.6	10	4.9
ACT			2	12.5	0	0	0	0	2	1
SA			1	6.3	2	1.7	4	6.6	7	3.4
NT			1	6.3	1	0.8	1	1.6	3	1.5
Tas			0	0	1	0.8	0	0	1	0.5
Total	9	4.4	16	7.8	118	57.8	61	29.9	204	100
**Market—Stories**										
**State**	**National**		**State**		**Regional**		**Suburban**		**Total**	
	***f***	***%***	***f***	***%***	***f***	***%***	***f***	***%***	***f***	***%***
Qld			3	4.7	158	41.3	77	46.1	238	37.5
NSW			5	7.8	136	35.5	42	25.1	183	28.9
Vic			18	28.1	77	20.1	33	19.8	128	20.2
WA			8	12.5	7	1.8	8	4.8	23	3.7
ACT			21	32.8	0	0	0	0	21	3.3
SA			8	12.5	2	0.5	6	3.6	16	2.5
NT			1	1.6	2	0.5	1	0.6	4	0.6
Tas			0	0	1	0.3	0	0	1	0.1
Total	20	3.2	64	10.1	383	60.4	167	26.3	634	100

**Table 4:** Ranked concepts for categories associated with Australian Magpie stories (newspaper markets).

Regional		Suburban		National		State	
Concept	Prominence	Concept	Prominence	Concept	Prominence	Concept	Prominence
Breeding	1.2	Swooping	1.6	Chick	6.6	Species	1.7
Season	1.1	Bike	1.4	Species	2.2	Birds	1.0
Nest	1.1	Area	1.4	Take	1.0	Take	1.0
Area	1.1	Attacked	1.4	Young	1.0	Year	0.9
Times	1.1	Protected	1.3	People	1.0	People	0.7
Protected	1.1	People	1.2	Birds	1.0	Season	0.7
Swooping	1.0	Times	1.1	Times	0.8	Attacked	0.6
People	1.0	Nest	1.1	Nest	0.8	Times	0.6
Birds	1.0	Season	1.0	Attacked	0.6	Nest	0.6
Attacked	1.0	Birds	1.0	Swooping	0.3	Swooping	0.3
